# Supplementary material for: Are carbon emissions trading and green financial instruments synergistic? -Comprehensive quantitative research based on content analysis
Source: PLoS One. 2024 Mar 7;19(3):e0298601. doi: 10.1371/journal.pone.0298601 (PMC10919601; doi:10.1371/journal.pone.0298601)
Supplement: S1 Data — (ZIP) [file pone.0298601.s001.zip › Data/地级市Z_F工作报告.zip!/╣≤╓▌/╣≤╤⌠╩╨/└┤╫╘╣1⁄2╓┌║┼í╛┬φ┐╦╩2╛▌═°í┐_2009.docx]

各位代表：

现在，我代表市人民政府向大会作工作报告，请各位代表予以审议，并请各位政协委员和其他列席人员提出意见。

一、2008年工作回顾

2008年，我们在省委、省政府和市委的正确领导下，在市人大、市政协的监督、支持和帮助下，以邓小平理论和“三个代表”重要思想为指导，全面落实科学发展观，深入贯彻党的十七大和十七届三中全会精神，紧紧围绕发展这个第一要务，千方百计创造条件加快发展，圆满完成了市十二届人大三次会议确定的各项目标任务，实现了建设生态文明城市的良好开局。全市实现生产总值811.1亿元，比上年增长13.1%，其中：第一产业增加值47.2亿元，增长7.2%；第二产业增加值381亿元，增长12.1%；第三产业增加值382.9亿元，增长15%。全市财政总收入完成224.3亿元，增长18.9%；地方财政收入完成89.1亿元，增长17.3%。全社会固定资产投资完成601.6亿元，增长20.2%。城市居民人均可支配收入达13816.8元，增长8.1%；农民人均纯收入达4817.8元，增长17.8%。居民消费价格指数同比上涨7个百分点。全市10个区（市、县）全部进入全省经济强县（区）行列。

（一）沉着应对复杂严峻形势的考验，努力促进经济又好又快发展

年初，我市遭受了百年不遇的凝冻灾害。大灾面前，我们按照市委的统一部署，全面动员抗灾救灾，确保了人民群众基本生活正常进行、城市正常运转和社会稳定。灾后及时启动“抓重建、保民生”工作，迅速恢复生产，大灾之年实现经济发展“目标不变、任务不减”的要求，夺取了抗灾救灾和恢复重建的重大胜利。

第三季度，百年不遇的国际金融危机对我市的影响日益加剧。我们沉着应对，危中寻机，迅速贯彻落实中央关于扩大内需保持经济平稳较快增长的政策措施，全力争取中央新增投资19.8亿元。11月起，新开工60个总投资近150亿元的项目，完成40个涉及资金共67亿元重大项目的竣工验收工作；同时，上报的一批重大项目被列为国家、省支持的重点项目。我市在迎接挑战、攻克难关中保持了经济持续快速增长，各级政府应对突发事件和驾驭复杂局面的能力得到增强。

（二）产业结构调整成效显著，综合经济实力进一步提升

服务业拉动作用进一步增强。实现社会消费品零售总额343.5亿元，增长23%，创历史新高。制定并实施《贵阳市进一步加快服务业发展的意见》及相关配套政策措施。大力发展旅游、会展、房地产、物流、金融、商贸、新兴服务业等七大重点领域，南国花锦、星力购物广场等20个大型商贸流通项目建成开业，完成合群路小吃一条街、箭道街美食广场、25个农贸市场和150个农家店升级改造，西南国际物流中心等一批物流重点项目开工建设。中信银行入驻贵阳，成为全省首家引进的股份制商业银行。成功举办贵阳避暑季系列活动，“爽爽的贵阳”、“避暑之都、温泉之城”城市品牌效应初步形成，旅游总收入达187.3亿元，增长51%。

工业结构进一步优化。工业增加值完成300.2亿元，增长8.4%。规模以上工业总产值达860亿元，净增105亿元。磷化工、铝及铝加工、现代中药、绿色食品、电子信息、装备制造等六大产业集群初具规模。高新技术产业发展步伐加快，启动贵阳高新技术产业经济带建设，斯奈克玛飞机发动机叶片生产线、飞舸电子元器件生产线等一批高新技术产业项目建成投产。中国·西部（贵阳）高新技术产业研发生产基地开工建设。自主创新能力逐步增强，完成重点技术创新项目50项，新建5家省级、16家市级企业工程技术中心。

农业综合生产能力不断提升。特色农业、城郊农业和高效农业进一步发展，奶牛、蔬菜、花卉、蛋鸡、药材等优势产业持续增长。投入1.5亿元新建10个标准化奶牛养殖小区，奶牛存栏量净增50%以上；蔬菜、鸡蛋、花卉等农产品实现自给自足并大量外销。病险水库除险加固、基本农田建设、万户小水池、农村危房改造、村寨串户路、四改一气建设项目全面完工，经过3年努力完成农村可建户用沼气池建设任务，基本实现全市农村普及沼气的目标。解决了20万农村群众的饮水安全问题。新阶段扶贫开发工作有序推进，减少农村贫困人口1.7万人。龙头企业带动作用突出，引进裕东、正大等一批知名龙头企业，新命名市级以上龙头企业20家。生态农业建设有序推进，启动乡村清洁示范工程18个。科技兴农和为农服务进一步加强，防汛抗旱、森林防火、重大动植物疫病防控形势总体平稳。

（三）重点项目建设进展顺利，城乡发展基础条件明显改善

全市纳入跟踪管理的74个重点项目建设达到预期目标，国际会议展览中心、高峰公司贵阳新基地二期等重点项目开工建设，开磷年产30万吨合成氨、华润雪花啤酒（贵州）公司22万吨/年生产线改扩建、贵阳海信平板电视产业化二期等项目建成投产，清镇80万吨氧化铝等重大技改项目步伐加快，清镇电厂异地技改、詹阳动力重工20—30吨液压挖掘机液压及电子控制系统等重点项目有序推进，西南环线一期竣工通车，白云污水处理厂、小河污水处理厂（二期）、开阳污水处理厂、金竹片区排污工程建成并投入使用，全力做好贵广快速铁路、厦蓉高速公路贵阳段等重大项目的协调服务。完成500公里农村公路硬化，建成7个乡镇客车站。

（四）资源节约和环境保护力度加大，生态建设成效显著

扎实开展以绿化营造林为重点的生态建设，恢复植被面积9.9万亩，完成营造林15.8万亩，全市森林覆盖率比上年提高1个百分点。节能减排工作取得新成效，关停和淘汰落后产能企业16户。单位生产总值能耗降低4.5%，二氧化硫削减3808吨，化学需氧量排放总量削减1155吨，城市污水处理率、城区生活垃圾无害化处理率分别达44%、92%，城市空气质量优良率为94.8%。实行严格的项目环评审批政策，拒绝289个涉及投资300多亿元的高耗能、高污染、低效益项目。循环经济建设稳步推进，实施重点项目25个。清洁生产、资源节约和综合利用取得明显成效，工业用水重复利用率和工业固体废物综合利用率大幅提高，矿产资源开发向规模化、集约化方向发展，土地节约集约利用和建筑节能等工作取得新进展。扎实推进“两湖一库”及南明河全流域综合治理，治理水土流失面积105.8平方公里。深入开展打击违法占用林地专项行动，严厉查处“福海生态园”等一批非法占用农用地、滥伐林木案件。

（五）城市建设管理水平显著提高，人居环境明显改善

启动贵阳市总体规划修编工作，编制完成二戈寨片区、小河区、三桥马王庙片区及其他重点区域控制性规划，城乡规划体系基本形成。将三桥马王庙片区行政管理体制整体划归云岩区管理。城市基础设施建设取得突破性进展，加快推进“三环路十六射线”城市骨干路网建设，环城高速公路、花溪二道、北京西路、油小线、贵金线、水东路、新庄污水处理厂等项目进展顺利。完成神奇路、市南路、新华路等城市主要道路“白改黑”工程及一批人行天桥建设，高质量完成中华路景观综合整治工程，建成3个山体公园。金阳新区建设步伐加快，奥体中心、观山公园开工建设，市妇女儿童活动中心、青少年活动中心等“文化山”项目有序推进，金元国际新城、金源世纪城等一批大型房开项目加快建设。着力提升城市精细化管理水平，在全省“整脏治乱”工作考核评比中获得第一名。城市美化、绿化、亮化、净化工作向街区、社区延伸，背街小巷环境明显改善，全市政府投资的518个公厕全部取消收费并向群众开放。大力清理各类违法建设，拆除违法建筑50万平方米，户外广告整治成效显著，城市总体形象有较大改观。大力实施“畅通工程”，达到全国B类城市一等管理水平。继续抓好城区地下管网建设和防洪排涝河道治理，城市防灾减灾能力增强。荣获“国家园林城市”、“全国创建文明城市工作先进城市”称号。

（六）各项改革不断深化，对外开放水平明显提升

大力推进行政审批制度改革，市级行政审批事项由625项减少到128项，成为全国省会城市中行政审批事项最少的城市之一。国有资产监管、国有产权转让得到加强和规范，完成40户国企改革改制工作。市属粮食企业全部完成改革改制任务并扭亏为盈。基本完成10余年来政府投资项目的“投转固”工作，为搭建投融资平台奠定了坚实基础。农村综合改革深入推进，集体林权制度改革基本实现明晰产权、落实经营主体的阶段性目标。科技、财政、人事、卫生、价格等领域的改革迈出新步伐。对外开放、招商引资取得重大进展。成功举办赴北京、重庆、长沙、武汉、上海、广州等地旅游推介和经贸交流活动。海关进出口总额完成22.5亿美元，增长33.1%。引进投资额5000万元以上大项目66个、世界500强企业1家。引进内资303亿元，增长20.2%；直接利用外资达9350万美元，增长15.4%。

（七）“六有”民生行动计划扎实推进，社会事业全面进步

为民办的十件实事如期完成。在全省率先实施义务教育均衡发展，稳步推进“两基”迎“国检”工作，农村标准化学校建设顺利推进，在全面落实国家对农村义务教育“两免一补”政策的基础上，对城市低保人群子女义务教育阶段实行了“两免一补”，确保所有进城务工人员子女接受义务教育。就业和社会保障水平进一步提升，鼓励城镇创业带动就业，新登记小企业和个体户分别为3817户和14461户，2549名下岗失业人员登记成为个体工商户。继续保持“零就业家庭”户数动态为零，充分就业社区达100个。新增就业岗位5.2万个，城乡统筹就业7.8万人，登记失业率控制在3.6%以内。大力开展帮扶返乡农民工就业创业百日活动，扎实做好农民工工资清欠工作。困难群众基本生活保障水平进一步提高，城镇基本养老、基本医疗、失业、工伤、生育保险参保人数分别达65.4万人、86.2万人、38.2万人、47.2万人和68.8万人，农村新型合作医疗、养老保险参保人数分别达161.1万人和4.2万人。中低收入家庭住房条件进一步改善，开工建设经济适用住房100.5万平方米，竣工107.7万平方米；开工建设廉租房10万平方米，竣工5.3万平方米，新增廉租住房保障家庭1937户；改造农村危房10670户。深入实施“严打两抢一盗，保卫百姓平安”专项行动，集中开展扫黄打非、禁毒等专项行动，社会治安进一步好转。成功处置“三鹿”问题奶粉及我市部分区域发生的甲肝疫情等食品卫生公共安全事件。安全生产形势总体稳定，实现事故起数和死亡人数“双降”目标。继续保持稳定低生育水平，全市人口出生率为10.5‰，人口自然增长率为5.44‰，符合政策生育率为97%，出生人口性别比回归正常值，人口与计划生育工作再获全省第一名。文化、体育事业稳步发展，全面实施农村广播电视“户户通”工程，成功举办全省首届农民运动会，圆满完成北京奥运会火炬贵阳境内传递活动，“爽爽的贵阳”大型交响音乐会等迎奥运进京演出活动获得圆满成功。连续第五次荣获“全国双拥模范城”荣誉称号。民族、宗教、档案、人防、外事、侨务、妇女、儿童、老龄、慈善、残疾人、红十字等各项事业取得新的进步。

（八）全面推进依法行政，服务型政府建设取得新成效

认真执行人大及其常委会的决议、决定，自觉接受人大及其常委会的法律监督、工作监督；积极支持政协和各民主党派、工商联、无党派人士参政议政，主动接受民主监督。办理人大代表建议154件、政协提案392件，办复率均达100%。坚持依法治市，深入推进“五五”普法依法治理工作。扎实开展“整治发展软环境，建设服务型机关”专项活动，着力解决软环境方面存在的突出问题，全面优化发展环境。组建市政务服务中心和为民办事全程代理中心，落实各项便民利民措施，绝大多数行政审批事项在窗口完成审批并办结。加强行政监察和审计监督，政府廉政建设取得新成效。

过去一年，我们兑现了对全市人民的承诺，取得的成绩来之不易，这充分说明了党中央、国务院、省委、省政府和市委的一系列重大决策是完全正确的，充分展示了“知行合一、协力争先”的贵阳精神风貌，充分体现了全市各级各部门驾驭复杂局面、领导科学发展、构建和谐贵阳的能力。在此，我代表市人民政府，向一年来辛勤工作、扎实苦干、团结拼搏、共创佳绩的全市广大干部群众，向驻筑解放军、武警部队、公安干警，致以崇高的敬意!向所有关心、支持贵阳建设和发展的各级领导、各界朋友，向各民主党派、工商联、无党派人士和人民团体，表示衷心的感谢!

在充分肯定成绩的同时，我们也清醒地看到，当前经济社会发展中仍然存在一些不容忽视的矛盾和问题，主要是：国际金融危机仍未见底，影响我市经济发展的不确定因素增多；经济结构调整和经济发展方式转变任务艰巨；农民持续增收的难度较大，解决“三农”问题的长效机制尚待健全；财政偿债还本付息压力较大，收支矛盾突出，引导社会资本参与经济社会建设尚需加大力度；社会保障体系有待进一步健全和完善，影响社会和谐的因素仍然存在等。对上述问题，我们将高度重视并认真加以解决。

二、2009年工作安排

今年工作的总体要求是:全面贯彻党的十七大、十七届三中全会、省委十届四次全会和中央、省经济工作会议以及市委八届六次全会精神，以邓小平理论和“三个代表”重要思想为指导，深入贯彻落实科学发展观，以促进社会和谐为根本目的，以加快生态文明城市建设为主线，应挑战、保增长、重民生、推改革、促开放、善领导，以创新驱动、项目带动、消费拉动为主要着力点和关键环节，促进经济发展方式转变和结构优化升级，变危机为机遇，化压力为动力，全力推动经济社会又好又快发展。

经济社会发展的主要预期目标是：全市生产总值增长12%，其中，第一产业增加值增长7%；第二产业增加值增长13%；第三产业增加值增长14%。财政总收入增长12%，地方财政收入增长12%。全社会固定资产投资增长25%以上。外贸进出口总额增长10%。社会消费品零售总额增长20%。城市居民人均可支配收入增长8%。农民人均纯收入增长10%。城镇登记失业率控制在4.5%以内。居民消费价格指数控制在4.5%以内。人口自然增长率控制在5.5‰以内。单位生产总值综合能耗降低4%，主要污染物排放减少2%。

实现经济增长12％的目标，既有挑战性，又有可能性。一方面，中央继续实行积极的财政政策和适度宽松的货币政策，加大投资力度，扩大国内需求，为我们提供了较前几年宽松的大环境。去年全市在遭受“两个百年不遇”重大灾害的极为不利形势下，经济增长的速度和质量都攀升到一个新的水平，我市夯实产业基础的成效将逐步显现。另一方面，贵阳经济基础还比较薄弱，经济总量还很小，如果我们在机遇面前仍然保持低速运行，与其他发达城市的差距将进一步拉大。我市的经济发展速度应该更快一点，才能不断提高广大人民群众的物质文化生活水平。当然，随着国际金融危机愈演愈烈，我市经济增长下行的风险客观存在，今年经济工作的难度较大。但只要我们坚定信心、知难而进，精心组织、扎实工作，就一定能实现预期奋斗目标。

为民办的十件实事为：

（一）为外来进城务工人员随迁子女接受义务教育提供免费教科书；为贵阳户籍市属中等职业学校学生免除学费。

（二）建立城乡临时救助制度；完善新型农村养老保险政策,2009年末全市新型农村养老保险参保人数达到10万人；新增城镇就业岗位4.5万个。

（三）改造19个乡镇卫生院；开工建设市妇幼保健院综合楼病房大楼、市口腔医院大楼。

（四）继续推进保障性住房建设，实施棚户区、工矿区危旧房改造工程；实施农村危房10000户改造工程；竣工经济适用住房100万平方米；建成廉租住房1000套5万平方米,将人均住房建筑面积不足15平方米的城镇低保家庭纳入廉租住房保障范围,做到应保尽保,实时保障。

（五）建成环城高速、贵金线、油小线、花溪二道、金朱东路、观山北路、奥体路、长岭南路等项目；开工建设贵阳北站；建成5座人行天桥；在中心城区新建3个山体公园（南明、云岩、小河各建1个）；完成奥体中心主体育场馆土建工程。

（六）建成新庄、修文、息烽、清镇百花湖、清镇站街污水处理厂及配套管网，城市生活污水处理率达80 %以上；基本建成花溪垃圾卫生填埋场；开工建设乌当垃圾焚烧发电填埋场。

（七）实施农村饮水安全工程,解决19.33万人饮水安全问题；实施800公里村寨串户道路硬化工程；实施20个乡村清洁工程；开工建设鱼洞峡水库。

（八）继续实施农村广播电视户户通工程，新增电视入户3.7万户，建成500个自然村广播站；建成164个村级综合楼；开工建设20个乡镇客车站；建设20家社区便民连锁超市；建成271个农家书屋,实现我市村村都有农家书屋。

（九）确保两湖一库水质恶化趋势得到初步遏制，启动两湖一库周边6个重点村寨农业面源和农村生活污染治理；完成2.6万平方米生态浮床和2个人工湿地建设；实施两湖一库周边退耕还林补植补造工程。

（十）继续实施一环四路综合管网入地二期工程，提高城市供电线网的运行安全质量，改善城市环境；提升城市数字化、网格化管理水平，实现城管和公安系统110、119、122三台合一的资源整合、信息共享。在互联网上开通平安民声服务台。

围绕上述要求、目标和任务，我们将着力抓好八个方面工作：

（一）以增加投资和扩大消费为重点，着力抢抓机遇保持经济平稳较快增长

千方百计促进固定资产投资大幅增长。抢抓中央扩大内需增加投资的机遇，坚持“建设一批、申报一批、储备一批”的项目工作思路，谋划一批事关民生福祉的民心工程、事关未来发展后劲的重大产业项目、事关城市竞争力和辐射力的重大基础设施项目、事关节能减排和环境保护的生态环境项目，及时更新项目库，确保全市重点项目库动态储备规模达2000亿元以上；按照国家投资方向，及时组织申报一批前期工作扎实的项目，抓好跟踪落实，力争更多项目列为中央、省重点项目，确保我市具备条件的“十一五”期间拟实施的重点项目全部在年内实施；抓好重大项目建设，加强对在建的中央、省、市重点项目的资金监管和质量监理，确保配套资金足额到位，保证工程进度和质量。加快推进科技含量高、带动能力强、符合产业政策、能实现更多就业和财税收入的重大产业项目建设，重点抓好开阳化工公司100万吨醇氨、贵州航天林泉电机公司复式永磁电机生产线、清镇80万吨氧化铝等产业化项目建设，完成贵州中烟工业公司异地技改项目。基础设施建设方面，配合省抓好贵阳至广州、贵阳至重庆、贵阳至成都快速铁路和贵阳至昆明、贵阳至长沙客运专线建设及龙洞堡机场改扩建等重大项目，加快推进贵阳铁路枢纽、厦蓉高速公路贵阳段、环城高速公路等工程建设，确保开工建设修文久长至开阳永温、清镇至织金等支线铁路和麦架至修文、息烽至开阳等高速公路，抓好开阳港、息烽港等港口及息烽至金沙大桥等通道的规划、建设，力争把贵阳建设成为大西南乃至西部地区的重要交通枢纽。配合省抓好黔中水利枢纽等重大项目，支持省电网公司完成30亿元电网建设改造投资。加快推进贵阳钢厂、贵州水泥厂、清镇电厂异地技改项目和鱼洞峡水库、华能焦化四号焦炉等重大关键性项目，改善基础条件。强化政府投资导向作用，整合优势资源，激活生产要素，促进民间资金更多地投向城乡基础设施、产业结构调整、公共事业等重要领域和薄弱环节。提高集约节约用地水平，统筹各业各类用地，合理安排城市建设用地、产业用地，预留发展空间。做好项目用地服务工作，确保国家、省、市级重大项目建设用地。采取积极有效措施盘活企业关闭、重组和改制中形成的存量建设用地。对使用原土地进行增资扩建的工业项目，不再征收土地价款，鼓励提高土地利用率和增加建设容积率。严格土地管理，落实土地开发整理和耕地占补平衡责任，成立市级土地储备中心，做好土地储备和土地出让工作，依法处置闲置土地，遏制土地违法行为。今年项目用地增加，征地、拆迁任务重、难度大，要坚持以人为本，落实好有关政策，做到和谐拆迁、依法拆迁。

想方设法努力扩大消费需求。继续提高城乡居民收入水平，着力解决企业职工、农民及退休人员、城乡低收入群众的增收和社会保障问题，加大对城镇低收入居民的扶持和救济力度，增加中等收入群体比重，健全城乡居民最低生活保障制度，完善教育、医疗服务体系，稳定群众消费预期，增强消费信心。认真落实促进农民增收的各项政策措施，建立农民工工资支付保障制度，提高农村居民消费能力，扩大农村消费。通过网络、媒体及政府、企业驻外办事机构及时对接、发布用工需求信息，多渠道扩大就业规模，帮助外出务工人员做好维权工作，通过增加就业提高城乡居民整体收入水平。在抓好保障性住房供应的基础上，全面放开房地产二级市场，鼓励个人贷款购房，扩大商品房消费，带动建材、装饰材料、家具、家电等消费市场的发展。进一步清理在汽车、建设、教育、医疗等领域的不合理收费，积极引导和促进家用轿车、电信、旅游、文化、体育、餐饮、休闲娱乐和其他服务性消费，举办各种展销会、购物节、房交会、美食节等，营造良好的消费环境，推进消费结构升级和总量扩大。继续推进“万村千乡”市场工程和“双百”市场工程，抓住国家推广“家电下乡”政策的机遇，帮助本地生产家电、食品、药品的企业争取进入采购范围。强化市场监管和物价监控，依法打击制假售假、虚假广告、商业欺诈等违法行为，加大价格监督检查力度，努力保持市场繁荣和物价总体稳定。

(二)以自主创新为突破口，着力推进产业结构优化升级

以自主创新促进产业结构优化升级。围绕建设创新型城市，进一步完善技术创新体系、知识创新体系和创新服务体系，推动经济发展由资源依赖型向创新驱动型转变。有效发挥财政资金的引导作用，逐步形成以企业为主体、市场为导向、产学研相结合的技术创新体系。整合省会城市创新资源，继续加强与省内外知名高校、科研院所的合作，重点支持创新平台建设，发展中介服务组织。实施特色优势产业创新提升计划，面向电子信息产业、装备制造业、特色食品、现代中药、化工等产业，实施一批带动力强的重大科技专项，建设一批产业技术创新联盟、工程技术中心和重点实验室，提升产业核心竞争力。深入实施品牌、专利和标准化战略，巩固发展国家知识产权工作示范城市创建成果，推行自主创新产品财政优先采购制度，支持企业参与制定并采用国际标准、国家标准和行业标准，提升贵阳产品整体形象。加强科普教育，提高市民科学素质。高新技术产业增加值增长25%；专利申请量增长20%以上，争创1个中国名牌产品；新增1个国家级、5个省级企业技术中心，新建2个公共技术平台。深入推进“人才强市”战略，加强人力资源市场建设，加大人才培养、引进、使用、激励工作力度，重点引进和培养高科技领军人才、高技能应用人才，切实提高我市对各类优秀人才的吸引力。

大力推进服务业提质增量。认真落实加快服务业发展的政策措施，推动服务业由消费型为主向生产型、消费型并重转变。积极引进培育各类金融机构，引进1—2家股份制银行或外资银行落户贵阳，继续做好市商业银行上市工作，加快组建农村合作银行，支持银行、证券、信托、保险等各类金融机构做大做强。重点完善基础设施、信息网络、物流配送三大平台，引进一批国内外知名物流企业，加快二戈寨物流园区建设，建成西南物流中心一期工程、扎佐物流园区战略装车点和贵州宝通工业物流园主体工程，发展第三方物流，加快构建高效物流体系，打造区域物流中心。提升信息服务业发展水平，加快数字内容产业园、白云动漫主题公园建设，软件业、动漫产业增加值增长50%。大力发展会展业，办好农博会、药博会、亚洲青年动漫大赛等一批大型展会，全力加快贵阳国际会议展览中心建设。规范发展法律咨询、会计审计、工程咨询、信用评估等商务服务业。下大力发展面向民生的服务业。加快商贸流通业现代化进程，培育1—2个销售收入超过10亿元的大型商贸企业集团，打造青云路步行一条街、白云夜郎古城美食街等特色商业街区，推进五里冲农副产品批发市场改扩建和元隆广场购物中心、中大国际购物中心、浙江商城二期等项目建设，新建和改造一批便民农贸市场项目，支持星力百货等商贸流通龙头企业建成社区连锁超市20家，方便居民消费。按照创建中国最佳旅游城市标准，举办好第二届避暑季和贵阳温泉节系列活动，彰显“爽爽的贵阳”和“避暑之都、温泉之城”品牌形象。加快凯宾斯基、希尔顿、世纪金源大酒店、保利温泉酒店等一批五星级酒店建设并促成开业，规范经济型酒店、乡村旅舍建设和发展，加强行业管理，提高我市旅游服务水平。精心组织第三届旅游产业发展大会和阳明文化节等系列活动，着力提高青岩古镇、保利温泉、天河潭、南江大峡谷、修文野生动物园、开阳十里画廊、息烽集中营等一批景区的知名度，下大力抓好我市重点旅游景区建设，形成避暑旅游、温泉旅游、红色旅游、乡村旅游、自然风光游等多层次旅游服务产品，实现旅游总收入增长50%。

扎实推进工业结构优化、集群发展和产业循环衔接。大力实施“工业强市”战略，积极为云马大客整车生产、万达客车新产品开发与生产、贵阳海信电子等终端产品生产企业提供政策、资金、用地支持，扩大市场竞争力和份额，支持贵州大力士轮胎公司、华工实业公司等配套零部件生产企业建立生产联盟，加强与上下游企业的联系，促进装备制造业整体提质增量。积极推进宝源阳光公司太阳能级多晶硅、振华新材料公司钴酸锂、钛易公司钛及钛合金等项目建设，打造我市新材料产业新优势。着力抓好开磷120万吨磷铵（二期）、中铝贵州公司20万吨电解铝、清镇80万吨氧化铝等项目建设，带动磷煤化工、铝精深加工配套项目建设，努力打造全国最大磷煤化工、铝加工循环经济示范园区。积极扶持益佰、同济堂、益康、新天、健兴等10家重点药业企业和20个重点药业品牌做大做强，将现代药业的总量和层次提高到一个新的水平。建成老干妈食品工业园，抓好五福坊、膳香坊、沁园及同济堂茶叶等食品企业生产线建设，促进食品工业提质增量。全市规模以上工业增加值增长13%，药业和食品工业的工业增加值增长20%以上。全面推动开发区“二次创业”。加快高新开发区新天园区经济提速和高新产业聚集。高新开发区拓展区以建设现代化、数字化、国际一流的新型高新技术园区为目标，以建设贵州航空高技术产业园为突破口，加快金苏大道、麦沙大道等园区主干道及水、电、气、动力等配套建设，开工建设5万平方米标准厂房，引进铝精深加工企业入驻园区，全面加快高新开发区建设步伐。充分发挥国家经济技术开发区体制机制优势，整合各方力量，加快小河—孟关装备制造业产业带基础设施建设，初步建成贵航军转民高新技术产业园，促进产业聚集。

（三）以城乡一体化为抓手，着力促进农业农村经济平稳较快发展和农民持续增收

坚持规划引领推动城乡空间布局一体化。高质量完成城市总体规划和土地利用规划修编，完成各区（市、县）总体规划、重点镇和村庄整治规划，加快城乡结合部和农村集中居住区等区域规划，形成城乡协调的空间结构体系、产业布局体系、基础设施体系、公共服务体系、生态环境体系，优化城乡空间布局，明确区域主体功能，实现城乡规划全覆盖。推动各区（市、县）重点工业园区、循环经济园区规划建设，促进工业向园区集中。加强小城镇、村庄整治和移民安置点规划，促进农民向集中居住区集中。继续推进城乡户籍一体化改革力度，为外来务工人员和常住人口及时办理城市户口，城市化率继续提高1个百分点以上。抓好农产品基地规划，引导农民集中连片发展种养业，促进土地向规模经营集中。

大力提升现代农业发展水平。继续实施“畜、禽、蛋、奶、果、蔬、花、药、茶、桑”十大现代农业示范工程，重点抓好4个标准化奶牛养殖小区建设，新引进奶牛2万头，使全市奶牛总量达到4万头，牛奶产量达12万吨；继续扶持三联、好一多等乳业龙头企业做大做强，从奶源生产、奶品加工到成品配送建立严格监控体系，打造生态奶、放心奶的贵阳乳业形象。鼓励边远山区、粮食产区推广种草养殖，建设生猪、蛋鸡规模化生产基地，改良品种、扩大规模，将清镇打造成全省最大的年出栏1200万羽肉鸡养殖基地，将开阳建成全省最大的年出栏50万头生猪生产储备基地。重点推进蔬菜、花卉、蚕桑、中药材种植等设施农业基地建设，提高农产品生产经营水平和产业发展实力。引进花卉物流企业，新增2000个花卉大棚，初步建立集科研、种繁、物流、营销、出口于一体的花卉发展体系。大力发展优良种苗业、高附加值加工销售业以及生态休闲农业，支持企业到郊县及外地建立基地和农产品销售网络，将贵阳建成种苗研发、繁育和农产品深加工、流通的集散地，形成“两头向外、两头在筑”的都市型高效农业发展模式。完善和拓展花溪、乌当国家级农业科技示范园区建设，努力把科技园区建成现代农业展示窗口。加强农产品质量安全体系建设，力争完成县级农产品质量安全检测和动物防疫体系建设。加强农业农村基础设施建设，抓好冬春农田水利基本建设及烟水配套工程建设，完成农业综合开发项目中低产田土改造3.39万亩、高标准农田基本建设3万亩，完成9座病险水库除险加固主体工程建设，新增灌溉面积1.9万亩，改善灌溉面积1万亩，在全省率先基本解决原规划确定的农村人口饮水安全问题。完善气象防灾减灾体系建设，提高公共气象服务能力。积极推进农村信息化工作，加快信息超市建设。加强县城及重点乡镇污水、垃圾收集处理设施建设，建成一批大型沼气池，完成贵遵公路贵阳段沿线等区域的村庄整治工作。按照新的扶贫标准，做好贫困乡村扶贫开发工作。

多渠道确保农民收入持续增加。大力发展劳务经济，在技能培训、就业创业、社会保障、户籍等关键环节大胆改革，扩大农村养老保险和大病医疗保险覆盖面，关心返乡农民工的就业和生计问题，切实解决农民工子女就学和农村留守儿童教育管理问题，不断增强农民就业创业能力，增加农民工资性收入。坚持依法自愿有偿原则，鼓励农村土地承包经营权流转，探索农村集体建设用地和宅基地使用权流转制度，增加农民财产性收入。实施好粮食收购、直补及良种、农机、农资等方面的补贴，确保资金落实到位，多渠道促进农民增收。

（四）以“喜迎民运会、创建国家文明城市”为城市建设管理主题，着力提升宜居宜业宜游城市品质

进一步扩大城市空间。大力实施“轻轨为轴，北拓南延西连东扩”城市空间拓展计划。力争轻轨和环城铁路开工，沿轻轨站规划城市建设空间、产业空间，以点——轴衍生模式拓展城市发展空间。按照建设高新开发区拓展区规划，加快麦架——沙文——扎佐片区基础设施建设和产业聚集，实现高新技术产业集群整体“北拓”。建成环城高速公路、花溪二道，通过“南延”加快花溪、小河等区域城市化进程和产业聚集。结合三桥马王庙片区发展规划，确保北京西路、贵金线和金朱西路建成通车，加快片区支路建设，通过“西连”三马片区促进老城区与金阳新区融合发展。按照建设临空经济区规划，以龙洞堡机场改扩建为契机，抓紧实施油小线建设，将中心城区与龙洞堡片区有机联结起来，整体启动龙洞堡——小碧——永乐片区建设，推进“东扩”建设东部新城。

努力提升中心城区品质。以实施“畅通工程”为重点，抓好城市交通基础设施建设，开工建设北二环、西南环线二期、北京东路等工程，建成水东路等中心城区出口通道，完成市北路至南垭路、青年路至水口寺及中坝路、210国道等损毁道路的维修改造，畅通城市交通内外循环系统。继续推进人行过街系统、无障碍设施、公厕等便民设施建设。推进“城中村”改造，启动大营坡片区和城市集中连片棚户区改造，新增城市绿地面积20万平方米。对宝山路、北京路、中山西路、瑞金路等城市主干道实施道路综合整治。

加快金阳新区开发建设。以新建的3条快速铁路和2条客运专线汇集金阳新区为契机，以抓好贵阳北站建设为切入点，推进金阳新区建设提速、产业聚集、管理创新、人气提升，金阳新区固定资产投资增长30%，新入住居民5万人。重点抓好金阳新区198平方公里总体规划、以贵阳北站为中心的17平方公里城区控规及中央商务区城市设计的编制，引进省内外大企业集团到金阳新区建设总部和研发中心，推动金元大厦、茅台大厦、开磷集团办公楼、贵州移动大厦、贵州联通大厦等10个总部大楼的建设，世纪金源25万平方米商贸城开业，建成金阳商业步行街、商业红街等一批商业项目并力争开业。全力推动金阳新区路网、综合管网、市政配套设施、园林景观等工程建设，启动金朱大道延伸到清镇的前期工作，抓好金朱西路、长岭路、观山北路、奥体路等新区道路建设和奥体中心主体育场建设，建成观山公园、青少年活动中心、妇女儿童活动中心，基本建成市级行政中心三期、工人文化宫，全面加快规划展览馆、博物馆建设。

着力提高城市管理水平。深化城市管理体制改革，以精细化、人性化管理为目标，全面启动数字化城市管理工作，对所有区实行标准统一的中心城区城市化管理模式。巩固发展“整脏治乱”成果，建立对“五脏五乱”等城市顽症的及时反应和快速处置机制，实现临时建筑“零申报”、违法建筑“零增长”。强化政府对土地的统一储备和一级市场培育、开发和管理，依法处置超期未开发的闲置土地，最大限度地盘活城市存量土地资源。鼓励支持社会力量投资公园、路桥、供水和垃圾处理等基础设施建设，大力推动环卫保洁、市政设施维护、园林绿化养护等公用事业市场化运作，面向社会有偿转让停车场、空间广告和公共设施冠名等公共资源经营管理权。力争实现创建国家卫生城市目标。

（五）以深化改革扩大开放为动力，着力构建有利于科学发展的体制机制

围绕重点领域和关键环节打好改革攻坚战。对市建设投资控股公司、金阳公司、市供水总公司等一批公司进行改革改制，组建文化旅游投资公司等一批符合现代企业制度、自主经营、自筹还贷、自负盈亏的投融资平台。强化国有产权管理基础工作，完善产权管理制度，推行国企资本经营预算管理和收益管理试点工作。继续推进国有企业改革改制、破产重组，全部终结政策性破产工作，完成50户国企改革重组任务。积极推动贵州华能焦化制气股份有限公司等一批企业上市。实施生态补偿机制，加快形成统一、规范、透明的财政转移支付制度。建立财政性投资项目“投转固”工作长效机制。

进一步扩大对外开放。改革创新招商引资方式，完善市、区招商引资利益激励分配机制，充分发挥商会、企业协会作用，形成互相协作、共谋发展的对外开放格局。切实优化招商引资结构，重点引进一批与我市产业链相衔接、与结构调整相融合的科技含量高、资源消耗少、对地方经济贡献大的项目。着力扩大对外交流领域，继续巩固与成渝、长三角、珠三角等区域的经济交流与协作，组织好赴韩国、台湾、北京、重庆、武汉等地招商引资、旅游商贸推介活动，力争引进1—2家世界500强企业、3—4家国内500强企业落户贵阳，在招商引资的规模和质量上实现突破。大力强化项目跟踪服务和检查，建立重点项目从洽谈、跟踪、签约、开工到竣工的全过程责任约束体系，提高项目签约成功率、资金到位率，招商引资到位资金增长20%以上。

加快非公有制经济发展步伐。进一步放宽非公经济准入范围和领域，积极引导和鼓励非公企业参与国有企业资产重组，进入城市基础设施建设、公共服务、旅游、农业、环保、高科技和文化产业等领域，提高非公经济在国民经济中的比重。实施“小巨人企业成长扶持工程”，大力发展中小企业融资公司，健全完善中小企业信用担保体系，发挥担保公司作用，鼓励通过股权融资、项目融资等方式筹集资金，支持中小企业培育名牌和技术创新。建立健全非公经济创业投资机制。依法保护非公资本投资项目和投资者的财产权、知识产权、自主经营权及其他合法权益。

（六）以建设资源节约型和环境友好型城市为目标，着力巩固和增强生态优势

大力推进循环经济发展。加快修订完成《贵阳市建设循环经济生态城市条例》，推动产业循环式组合、企业循环式生产、资源循环式利用。继续推进清镇煤化工示范基地、开阳磷煤化工生态工业示范基地、息烽磷煤化工生态工业基地、白云铝及铝加工基地、修文医药园区建设。加快城市再生资源回收利用体系建设，继续实施好中化开磷2×5亿块磷渣砖、紫江水泥公司磷渣综合利用年产120万吨熟料、青利公司黄磷尾气综合利用年产7万吨甲酸钠、城区泔水油统一回收加工利用等一批循环经济项目。

大力推进生态环境建设。继续开展荒山造林、退耕还林、石漠化治理试点、天保工程公益林建设，建设和保护好二环林带，加大打击违法占用林地专项行动工作力度，新增营造林面积12万亩，确保全市森林覆盖率提高1个百分点。依法加强矿产资源的管理与保护，规范资源开采和综合利用，建立完善资源有偿使用和生态补偿制度。切实加强环境保护和污染治理，继续加大“两湖一库”等重要水源地保护与治理力度，启动环湖林带建设，完成集中式饮用水源保护区地理界线的实地标定，实现水质稳定好转的目标。加大小关湖、黔灵湖治理力度，使水体水质达到人体可接触标准。抓好南明河全流域综合治理，启动花溪十里河滩湿地公园建设，促进城市生态环境进一步改善。

大力推进节能减排。加大对现有耗能工艺、设备的技术改造力度，关停浪费资源、污染严重的企业，淘汰落后生产工艺和设备。加强重点行业、企业的能耗、物耗管理，建立清洁生产绩效评估制度，积极推进钢铁、有色、化工、建材、电力等重点耗能行业和企业的节能工作。严格控制大气污染物的排放，全面启动机动车尾气监测工作。加快危险品及固体废弃物处置中心等项目建设。建成新庄污水处理厂及一批重点城镇污水处理厂和管网收集系统，使污水处理能力达到80%。大力推进农村环境保护，切实防治农业面源污染。

（七）以“六有”民生行动计划为主要途径，着力构建和谐贵阳

围绕“学有所教”工作目标，合理配置教育资源。确保“两基”通过“国检”，大力推进区域内义务教育均衡发展，继续抓好“两免一补”和免除城市义务教育学杂费等政策的落实，着力解决“上学难、上学贵”问题，促进教育公平。继续拓展高中阶段教育，改造城区薄弱初中学校，扎实推进贵阳二中、六中、民族中学改扩建工程，积极接纳外来务工人员子女接受公办义务教育。抓好中小学布局调整规划，稳步推进农村中小学标准化建设，力争完成17所标准化学校建设。办好贵阳学院，不断提高教育教学质量，做好迎接教育部本科教学评估工作。大力发展职业技术教育，完成贵阳护理职业学院搬迁，基本建成贵阳职业技术学院，打造一批具有优势特色专业的国家级、省级重点中等职业学校。强化师德师风教育，抓好师资队伍建设。

以“劳有所得”为重点，努力提高城乡居民收入。完善和落实小额贷款、税费减免等就业政策，鼓励劳动者自主创业和自谋职业，多渠道开发、增加就业岗位，切实做好就业再就业工作。完善困难群体再就业援助制度，增加公益性岗位，保持“零就业家庭”动态为零，30%的社区建成“充分就业社区”，确保实现城乡统筹就业6.8万人，城乡统筹培训4.5万人。

以“病有所医”为切入点，大力推进公共卫生服务体系建设。抓好医疗保险向所有城镇扩面工作，全面推行城乡医疗救助制度。加强惠民医疗服务，确保惠民医疗政策100%覆盖城乡低保户、五保户等困难群众。巩固和完善新型农村合作医疗制度，确保农业人口参合率达95%，基金使用率达85%以上。积极推进城市社区卫生服务工作。组建市康复疗养基地。加强结核病、艾滋病防控工作，全面落实重大疾病和高致病性人畜（禽）共患疾病的预防控制措施，严防重大疫情发生和传播扩散。继续稳定低生育水平，大力提高出生人口素质，扎实抓好出生人口性别比综合治理工作，完善计划生育“奖、扶、医、帮、优、保”系列利益导向政策机制体系，进一步加强流动人口计生服务管理，全面提升人口计生工作整体水平。

以“老有所养”为突破口，进一步完善社会保障体系。加大社会保险扩面征缴力度，强化社保基金监督管理，实现养老保险扩面5万人。继续推进养老、医疗、失业、工伤、生育保险和社会救助体系建设，积极探索解决老年居民养老保障问题。

实施“住有所居”行动计划，切实解决城乡低收入家庭住房困难。落实好国家促进房地产市场健康、有序发展和刺激住房消费的若干政策，优化住房供给结构，满足群众对小户型、中低价位商品住房的需求，规范经济适用住房建设、销售政策，加大廉租房建设力度，抓好农村危房改造工作，进一步健全住房保障制度。

大力推进“居有所安”工程，切实维护社会稳定。加强社会治安综合治理，完善社会治安防控体系，深入开展“严打两抢一盗，保卫百姓平安”专项行动，抓好“扫黄打非”工作，深入贯彻《禁毒法》，全面推广社区禁毒工作，实现修文、息烽、开阳等县创建无毒县的目标，妥善解决群众反映强烈的社会治安问题。认真做好信访工作，建立完善矛盾调处和社会利益协调机制，从源头上预防和减少矛盾纠纷。切实加强人民调解、行政调解工作，及时疏导和化解矛盾纠纷。严格落实安全生产责任制，加大煤矿、道路交通、消防等重点行业的安全生产检查力度，对存在重大安全隐患的生产经营场所，坚决予以关停整顿，最大限度地控制和减少各类事故发生，确保事故起数和死亡人数控制在省下达指标范围内，进一步加大地质灾害防治力度，保证人民群众生命财产安全。加快食品质量检验检测中心建设，继续强化对食品、药品在生产、经营、使用等各环节的监管，确保公众的饮食、用药安全。

大力推动文化、广电和体育事业发展。深化文化和广播电视体制改革，积极稳妥推进以市属经营性文化事业单位和专业艺术院团为重点的文化体制改革，大力发展文化产业。举办好第七届中国舞蹈“荷花奖”民族民间舞大赛，完成阳明书院的建设，加强县乡文化馆（站）、社区文化活动中心阵地建设，积极推动文化事业繁荣发展。全力推进“两台一站”数字化，完成有线电视数字化平移5万户和农村电影放映1.4万场。加快推进体育事业发展，精心举办好贵阳市第八届民运会，积极做好全国第九届民运会的相关前期准备工作，加强体育场馆等设施的建设和管理，开工建设市民健身中心,大力开展全民健身运动。

（八）以建设服务型政府为目标，着力打造一流政务服务环境

加快转变政府职能，提高政府服务质量和效能。各级政府要始终坚持以发展为第一要务，不动摇、不懈怠、不折腾，在加强和改善经济调节、市场监管的同时，更加注重社会管理和公共服务，确保政府管理、服务到位。深入推进政企分开、政资分开、政事分开、政府与市场中介组织分开，促进行业协会、中介机构规范发展，推动公益类事业单位和行业协会、中介机构更多、更好地承担行业管理和公共服务职能。进一步提高政务服务中心和为民服务全程代理中心服务水平，强化并联审批机制，加快网上审批和在线审批系统建设，改革部门内部的审批工作机制，使政务服务中心真正成为“审批服务窗口”，打造一流的综合政务服务平台。坚持政务信息“公开是常态，不公开是例外”的原则，健全政务公开制度、新闻发布制度和重大突发事件报道快速反应机制，最大限度地保障群众的知情权、参与权、表达权和监督权。以深化财政支出管理改革为重点，健全公共财政框架。集中财力办大事、做实事，财政支出以保增长、保民生、保运转为重点，千方百计降低行政成本，确保实现全市行政公用经费、行政购车经费、出国考察经费“零增长”的目标，将新增财力主要用于改善民生、公共基础设施建设和“三农”等方面。

严格依法行政，确保行政行为规范有序。深入推进依法治市工作，增强政府依法行政能力。认真落实人大及其常委会各项决议决定，坚持向人大及其常委会报告工作，自觉接受监督。主动加强同政协的联系并向其通报工作，支持政协委员发挥参政议政作用。提高建议、提案的办理时效和质量。广泛听取各民主党派、工商联、无党派人士意见，支持工会、共青团和妇联等人民团体更好地发挥作用。深入推进村（居）民自治，巩固和扩大基层民主。

树立优良政风，建设廉洁政府。加强公务员队伍教育、培训和管理，使广大公务员特别是领导干部牢固树立并始终坚持正确的事业观、工作观和政绩观，保持昂扬向上的精神状态、开拓进取的工作状态、勤政为民的服务状态，树立人民公仆的良好形象。坚持问政于民、问需于民、问计于民，完善重大决策专家咨询和社会公示、社会听证制度，真诚倾听群众呼声，真实反映群众意见，真情关心群众疾苦，按照“群众生活中的小事，就是政府工作的大事；群众生活中的难点，就是政府工作的重点”的要求，努力为群众办好事、办实事、解难题。扎实推进惩治和预防腐败体系建设，在坚决惩治腐败的同时，更加注重治本，更加注重预防，更加注重制度建设，健全建筑工程、土地交易、产权交易、政府采购、资金监管等重点领域的反腐长效机制，从源头上防治腐败。把依法管理、严格监督、透明运行贯穿于财政资金运作的全过程、各环节，扩大财政资金绩效审计，提高财政资金使用效益。深入推进“整治发展软环境，建设服务型机关”专项活动，加强政风行风建设，切实改善发展软环境。整合政府监督资源，充分发挥审计、监察机关作用，推进电子监察系统建设，扩大电子监察在政府工作监督中的覆盖面，强化对政府部门履职情况的监督问责，确保政令畅通。加强政府督查工作，完善政务督查信息网络，提高督查时效。

各位代表，2009年将是我市经济社会发展不平凡的一年，我们面临的形势更加严峻，承担的任务更加繁重，肩负的责任更加重大。我们要按照胡锦涛总书记对贵阳市“做表率、走前列”的要求，认真贯彻落实中央、省经济工作会议精神，按照省委十届四次全会、市委八届六次全会的总体部署和要求，以科学发展观统揽全局，抢抓机遇，奋发有为，开拓进取，扎实工作，为加快生态文明城市建设作出新的更大的贡献！
